# Twenty years of research on exercise-induced fatigue: A bibliometric analysis of hotspots, bursts, and research trends

**DOI:** 10.1097/MD.0000000000041895

**Published:** 2025-03-21

**Authors:** Qiwen Xuan, Lele Huang, Wei Gu, Changquan Ling

**Affiliations:** a School of Traditional Chinese Medicine, Naval Medical University, Shanghai, China; b Department of Sports Medicine, Peking University Third Hospital, Institute of Sports Medicine of Peking University, Beijing, China.

**Keywords:** bibliometric analysis, CiteSpace, exercise-induced fatigue, VOSviewer

## Abstract

Data from the Web of Science Core Collection (2004–2023) on “exercise-induced fatigue” were analyzed using bibliometric tools to explore research trends across countries, institutions, authors, journals, and keywords. The analysis was limited to “Article” and “Review” literature types. Among 4531 publications, the United States contributed the most articles (1005), followed by England (559) and China (516). The most influential institution was Universidade de São Paulo, while the most productive was Institut National de la Santé et de la Recherche Médicale with 103 papers. The European Journal of Applied Physiology ranked as the top journal with 233 articles. Millet Guillaume Y. emerged as the most prolific author, and Amann Markus was the most cited. Recent keyword trends showed a surge in terms like “physical activity” and “aerobic exercise,” while “fatigue” and “exercise” remained dominant. Notable findings were observed in oncology, engineering, and multidisciplinary studies, indicating potential research trends. Oxidative stress was identified as the most commonly mentioned mechanism in exercise-induced fatigue studies. This bibliometric analysis highlights current research trends and gaps, suggesting that future studies should focus on understanding the mechanisms of exercise-induced fatigue, developing objective measurement criteria, and exploring strategies for its alleviation.

## 1. Introduction

Exercise-induced fatigue, characterized by the inability to initiate or sustain voluntary activity following intense and prolonged exercise,^[1]^ often results in decreased motor function, reduced work efficiency, and increased risk of accidents.^[2]^ Prolonged fatigue can also elevate the incidence of various diseases such as multiple sclerosis, Parkinson disease, and depression, significantly impacting patients’ work and daily life.^[3–5]^ Exercise-induced fatigue is categorized into physiological and psychological fatigue. The biological mechanism of exercise-induced fatigue is not yet fully understood and is related to various factors such as changes in central neurotransmitters, imbalance of energy metabolism, disturbances in the internal environment and neuromodulation, oxidative stress and inflammation induced by exercise stress.^[6,7]^ In recent years, due to the great practical significance of sports fatigue research, many scholars at home and abroad are committed to this field and the research results are fruitful, in recent years, the academic output is with the development of science and technology is increasing year by year, sports, military and aerospace and other sports fatigue concerns and research gradually in-depth, how to eliminate fatigue has been the hot spot of the research.^[8]^

Bibliometric analysis, widely utilized across diverse disciplines, quantitatively and qualitatively assesses published academic literature within a specific research domain, providing an objective overview of the current research landscap. The software tools commonly used in bibliometrics are CiteSpace and VOSviewer. These tools can generate visualizations of country distribution, collaborative networks of research institutions, author networks, keyword co-occurrence patterns, keyword clusters, and reference co-citations.^[9]^ In recent years, many scholars of basic medicine and clinical medicine have carried out bibliometric research on diseases.^[10–12]^ Due to their power, user-friendliness, flexibility, and widespread acceptance in academia, these tools have gained significant recognition.^[13,14]^ CiteSpace, developed by Professor Chen Chaomei of Drexel University, automatically import data and analyze research trends to generate informative visual knowledge maps.^[15]^ VOSviewer, developed by Nees Jan van Eck and Ludo Waltman at Leiden University in the Netherlands, features a user-friendly interface, clear graphical representation and high accuracy.^[16]^

Considering that the research on exercise-induced fatigue has already had a certain foundation and developed rapidly in recent years, it is imperative to explore its historical progress, understand the contributions and disparities among different countries and regions, and anticipate future research trends. However, no bibliometric analysis of the global literature on exercise-induced fatigue has been conducted to date. This study aims to perform a comprehensive bibliometric visualization analysis of studies on exercise-induced fatigue published worldwide over the past 2 decades (January 2004–December 2023), in order to elucidate the current research status, identify prevalent issues in this field, and offer valuable insights for future research endeavors.

## 2. Methods

### 2.1. Search strategy

On February 14, 2024, the search was conditional on the title containing “exercise-induced fatigue” and its subheadings in the Web of Science Core Collection, Science Citation Index Expanded with publication timespan from January 2004 to December 2023. In order to improve the quality of the study, the literature types were limited to “Article” and “Review,” and the language was set to “English.” A total of 4531 articles meeting these criteria were retrieved. These articles were subsequently downloaded into both CiteSpace and VOSviewer for detailed visual analysis.

Due to potential limitations in associating certain cities with their respective countries and variations in institution names, an automated consolidation process was implemented to ensure accuracy in attributing countries and institutions. Similarly, synonymous keywords and authors with differing abbreviations were unified. A manual disambiguation process was further undertaken for country, research institution, and author data. Keywords with similar meanings were merged for clarity. For instance, “England,” “Northern Ireland,” “Scotland,” and “Wales” were grouped under “United Kingdom.” Additionally, variations in institution names such as “Franklin Pierce Coll” and “Franklin Pierce Univ” were combined under “Franklin Pierce Univ.” Similarly, variations like “ariens, ga” and “ariens, gam” were unified as “ariens, gam.” Consistency was maintained by merging terms like “skeletal-muscle” into “skeletal muscle,” and so forth.

### 2.2. Bibliometric analysis and visualization

CiteSpace 6.3 R1 Advanced Edition was primarily employed in this study to identify burst keywords across various research categories and construct time zone maps. The settings in CiteSpace were configured as follows: Time Slicing was set to 1 year, the association strength algorithm was determined as “Cosine,” and TOP N was set to 25. The Nodes were sequentially set as institutions, keywords, subject categories, and journals, while Pruning was applied using both “Pathfinder” and “Pruning to Network” methods.

VOSviewer 1.6.19 was utilized to explore co-occurrence relationships among countries, journals, keywords, and articles. In the visual representations generated by these tools, each node represents a research entity, with node size indicating frequency of occurrence in the study. The connections between nodes signify the strength of association.^[14]^ In VOSviewer, the type of analysis was set to Co-authorship, Co-occurrence, or Citation, with Units of analysis selected as Journals, Authors, Organizations, All Keywords, and Documents. Additionally, Microsoft Excel was employed for overall data analysis, and Scimago Graphica 1.0.36 was utilized for map visualization.

## 3. Results

### 3.1. Analysis of publications

**Figure 1.**
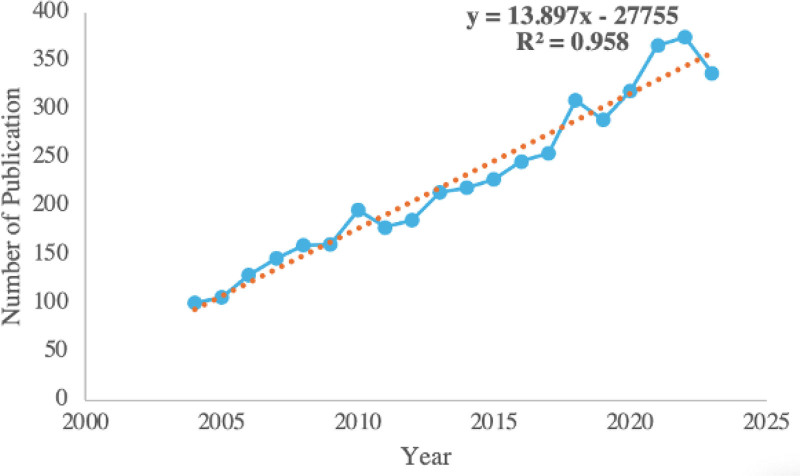
Number of annual publications on exercise-induced fatigue from 2004 to 2023.The number of published documents increases exponentially, and conforms to the formula: *y* = 13.897 × –27,755, *R*^2^ = 0.958.

The analysis of academic literature on exercise-induced fatigue over time reveals significant trends and patterns, offering insights into the research status and future directions of this field. A comprehensive examination of 4531 publications, comprising 4032 articles and 499 reviews, was conducted. Figure 1 illustrates a notable increase in the number of publications from January 2004 to December 2023. The data indicates a consistent upward trajectory, with an average of 226.55 articles published annually, representing a 3.35-fold increase since 2004. Notably, 44.10% of the articles were published after 2018, reflecting a recent surge in research activity. The peak occurred in 2022, with 375 publications. The growth of publications on exercise-induced fatigue follows an exponential trend, as evidenced by the predictive model formula: *y* = 13.897 × −27,755, with an *R*^2^ value of 0.958, indicating a well-fitted curve. This suggests a strong research foundation and increasing scholarly interest in this field. However, the subsequent decline in publications after 2022 signals the need for breakthrough progress, encouraging the exploration of new research avenues and emerging hotspots. The observed growth curve aligns with the logical trajectory of bibliometric analysis, underscoring the importance for researchers to continuously track real-time data to stay abreast of cutting-edge research and identify opportunities for innovation within the field of exercise-induced fatigue.

### 3.2. Analysis of countries/regions

**Figure 2.**
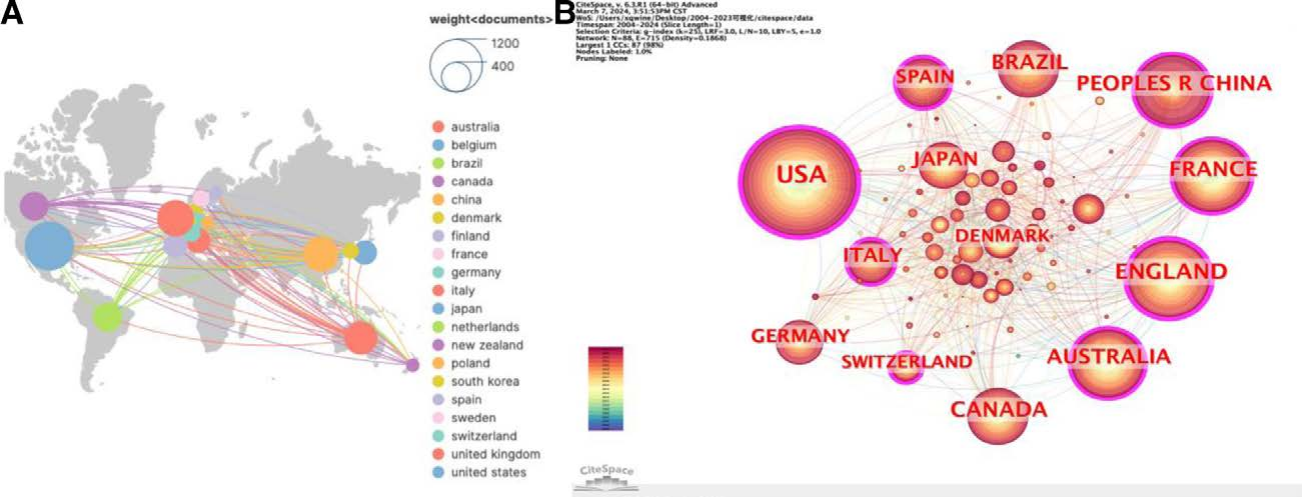
Analysis of countries/regions: (A) cooperation map of countries from 2004 to 2023. (B) map of country or regional cooperation networks.

We analyzed the countries and institutions of publications including a total of 88 countries/ regions, and the cooperation between them constituted 715 links (Fig. 2). In Table 1, The United States emerged as the leading contributor in this field, accounting for the largest number of papers (1005 publications, 22.18%), followed by England (559 publications, 12.34%), China (516 publications, 11.39%), France (471 publications, 10.40%), and Australia (454 publications, 10.02%). In Figure 2A, the size of each circle corresponds to the number of papers published by the respective country/region, with larger circles indicating a higher volume of publications. Additionally, links between countries represent collaboration networks. Figure 2B depicts a map of national or regional cooperation networks, with the outermost purple circle denoting centrality. A higher centrality score indicates greater importance of the node within the network. The data underscores the dominant role of the United States in terms of publications, citations, and international collaboration in the field of exercise-induced fatigue. Furthermore, France stands out for having the earliest average publication time, signifying its pioneering efforts in this area of research.

**Table 1:** Top 10 countries with the highest number of articles on exercise-induced fatigue.

Rank	Country	Links	Publications	Citations	Avg. citations	Avg. publication yr
1	United States	51	1005	38,612	38.4199005	2015.06
2	England	50	559	21,251	38.01610018	2015.49
3	China	32	516	8494	16.46124031	2018.62
4	France	37	471	15,298	32.47983015	2014.85
5	Australia	42	454	15,728	34.64317181	2016.52
6	Brazil	33	351	6905	19.67236467	2016.97
7	Canada	38	345	10,834	31.40289855	2015.26
8	Italy	42	260	8184	31.47692308	2016.78
9	Japan	22	246	5166	21	2015.05
10	Spain	39	244	6004	24.60655738	2017.48

### 3.3. Analysis of institutions

**Figure 3.**
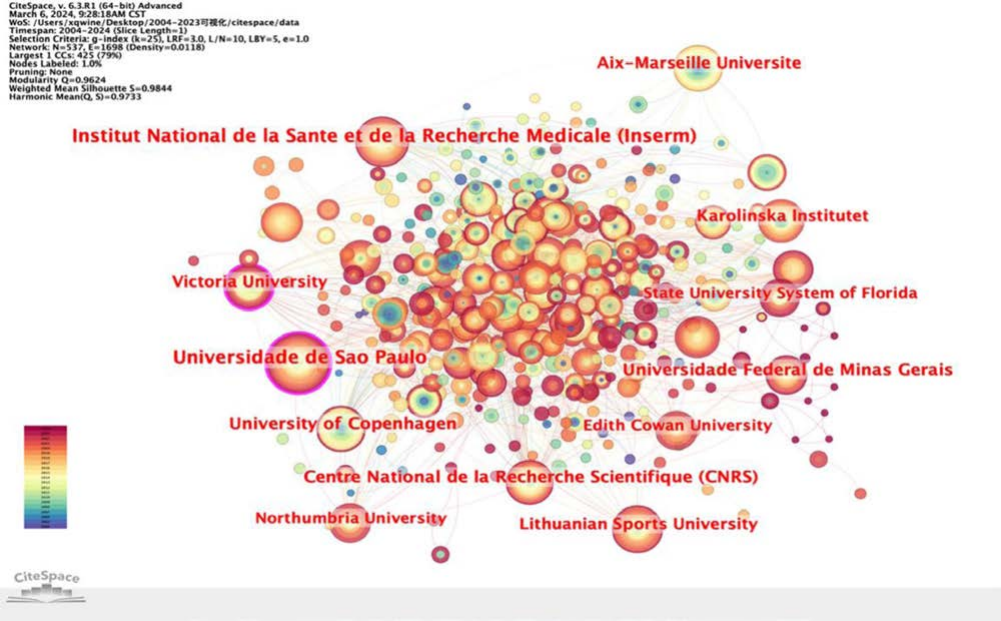
Analysis of institutions: cooperation map of institutions from 2004 to 2023.

A total of 537 institutions and 1698 connections were identified, with a network density of 0.0118, as illustrated in Figure 3. Nodes with high centrality serve as significant focal points within the field, represented by the purple outer circle in the figure. The width of the circle indicates the degree of centrality.^[17]^ The lines between nodes signify cooperative relationships,^[18]^ with a greater number of lines indicating more extensive collaboration. Institutions such as Universidade de Sao Paulo and Victoria University, with centrality values exceeding 0.1, hold prominent positions in the field and have established robust research collaborations with other institutions (Table 2). Ranking by the number of articles published, the top 5 institutions include Institut National de la Sante et de la, Universidade de Sao Paulo, University of Copenhagen, Recherche Scient fique, Universidade Federal deMinasGerais, Victoria University. They are from 5 different countries. Notably, there are close connections between these institutions, highlighting the prevalence of collaborative efforts. Most of the identified institutions are universities, underscoring their pivotal role as breeding grounds for scientific researchers and the generation of significant research outcomes.^[19]^ This underscores the importance of increasing investment in research institutions and hospitals to foster further scientific advancements.

**Table 2:** Top 6 institutions with the highest number of articles on exercise-induced fatigue.

Rank	Institutions	Countries	Citations	Documents	Avg. citations	Centrality
1	Institut National de la Sante et de la	France	4553	103	44.20	0.05
2	Universidade de Sao Paulo	Brazil	3119	94	33.18	0.17
3	University of Copenhagen	Denmark	4021	73	55.08	0.08
4	Recherche Scient fique (CNRS)	Canada	2727	73	37.36	0.06
5	Universidade Federal deMinasGerais	Brazil	1086	66	16.45	0.03
6	Victoria University	Australia	3175	65	48.85	0.11

### 3.4. Analysis of authors

**Figure 4.**
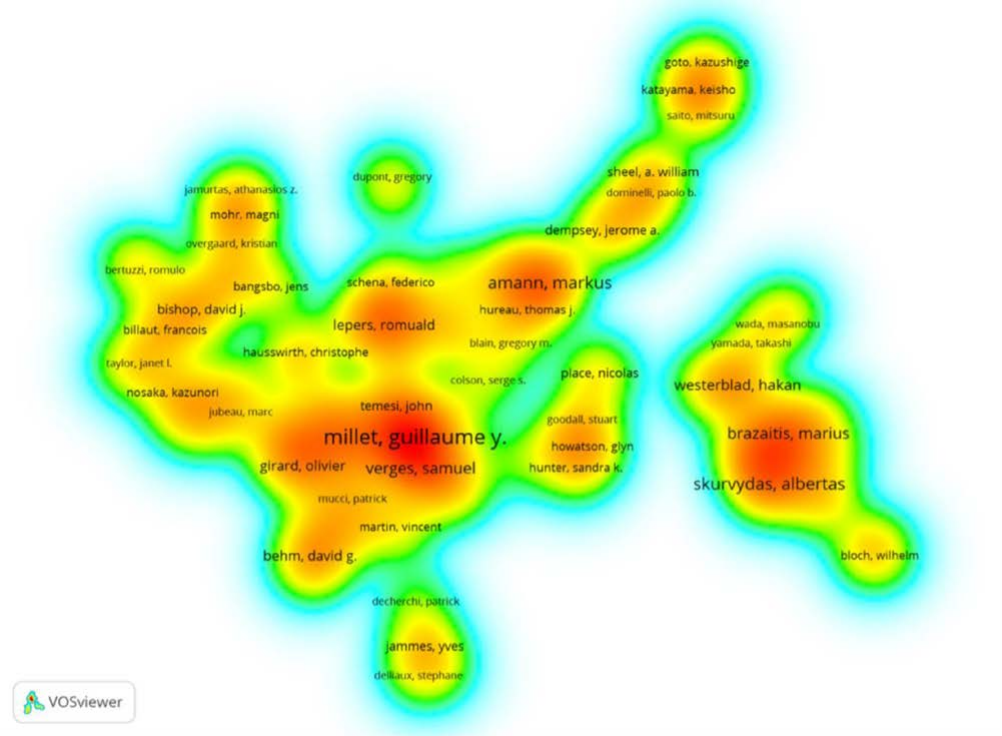
Analysis of authors: density visualization.

For improved visualization, only 91 authors were included in the collaborative author network, all meeting the minimum requirement of having more than 9 articles. In the knowledge graph, nodes represented authors, and links denoted relationships. Figure 4 displayed 5 different colors, with the red color indicating a higher author density index, as observed in the density visualization.^[20]^ This visualization method mirrored the distribution of identified keywords based on their frequency of appearance.^[21]^ In Table 3, we presented the top 10 authors with the highest number of articles from 8 different countries. Among them, the author with the strongest collaborative connections and the highest publication output was Millet Guillaume Y, affiliated with Institut Universitaire de France. His research spanned across Sport Sciences, Physiology, Neurosciences and Neurology, General and Internal Medicine, and Science & Technology. Notably, his most cited review, titled “Alterations of neuromuscular function after prolonged running, cycling and skiing exercises,” received 264 citations.^[22]^ This review provided a comprehensive analysis of the mechanisms underlying muscle fatigue following prolonged aerobic exercise, encompassing both central and peripheral aspects. The h-index, utilized to quantify a researcher’s scientific output and citation impact, was obtained from Google Schola.^[23]^ The author with the highest h-index score was Hakan Westerblad, affiliated with Karolinska Institutet. His research spanned Physiology, Neurosciences and Neurology, Cell Biology, Biochemistry & Molecular Biology, and Sport Sciences. While the articles by Markus Amann garnered the highest number of citations, their total link strength was not high, suggesting the need for enhanced collaborative communication with other researchers. His most cited article, titled “Opioid-mediated muscle afferents inhibit central motor drive and limit peripheral muscle fatigue development in humans,” was cited 311 times.^[24]^ This study conducted experiments that selectively blocked lower limb afferent feedback during whole-body exercise in humans without compromising the force and power-generating capacity of the locomotor muscles. It underscored the role of locomotor muscle afferent feedback in limiting the development of peripheral exercise fatigue up to a critical threshold during high-intensity whole-body endurance exercise.

**Table 3:** Top 10 authors with the highest number of articles on exercise-induced fatigue.

Rank	Authors	Institutions	Countries	H-index	Citations	Documents	Avg. citations
1	Millet Guillaume Y.	Institut Universitaire de France	France	53	2063	45	45.84
2	Amann Markus	University of Utah Dept Anesthesiol	United States	43	2492	31	80.39
3	Skurvydas Albertas	Vilnius University	Lithuania	25	713	30	23.77
4	Wanner Samuel P.	Universidade Federal de Minas Gerais	Brazil	19	480	29	16.55
5	Brazaitis, Marius	Lithuanian Sports University	Lithuania	21	462	29	15.93
6	Westerblad Hakan	Biomedicum Karolinska	Sweden	67	915	24	38.13
7	Coimbra Candido C	Universidade Federal de Minas Gerais	Brazil	30	825	21	39.29
8	Girard Olivier	University of Western Australia	Australia	41	1263	21	60.14
9	Behm David G.	Memorial University Newfoundland	Canada	10	418	19	22
10	Meeusen Romain	Vrije Universiteit Brussel	Belgium	35	1032	17	60.71

### 3.5. Analysis of keywords and strongest burst keywords

**Figure 5.**
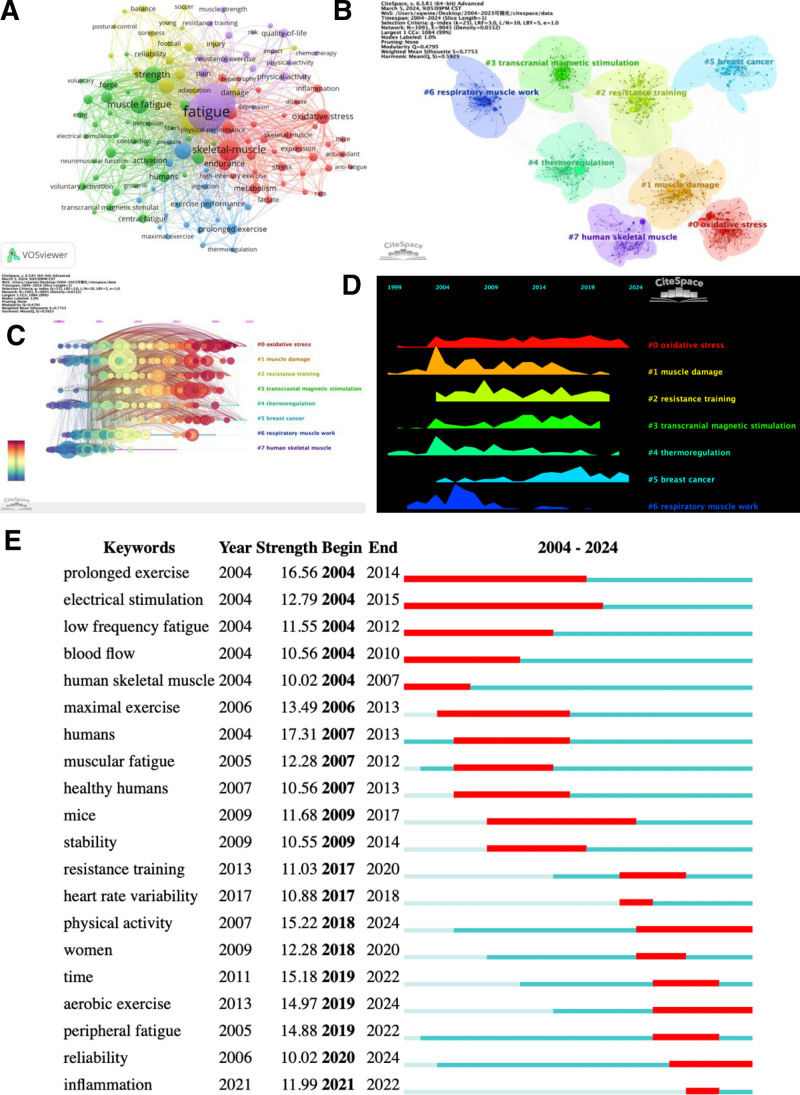
Analysis of keywords: (A) keyword co-occurrence. (B) Cluster analysis of keywords. (C) Timeline chart of keywords (D) Keywords Landscape view of timeline chart (E) Top 20 keywords with the strongest citation bursts.

Using keywords as nodes, a total of 13,893 keywords were analyzed, with only 132 keywords appearing more than 50 times (Fig. 5A). Notably, “fatigue” (1628 occurrences) and “exercise” (1542 occurrences) surpassed 1000 occurrences, emerging as the 2 most central words. Other prominent research focuses include muscle fatigue (437 occurrences), skeletal muscle (603 occurrences), and performance (818 occurrences), indicating the close relationship between the study of exercise-induced fatigue and muscle fatigue, particularly skeletal muscle fatigue. Log-likelihood ratio clustering was performed on the included keywords, and 8 clustering modules were identified (Fig. 5B). These modules correspond to the following topics: #0 oxidative stress, #1 muscle damage, #2resistance training, #3transcranial magnetic stimulation, #4 thermoregulation, #5 breast cancer, #6 respiratory muscle work, and #7 human skeletal muscle. The Q value, which represents the clustering module value, was found to be 0.4795. A Q value >0.3 indicates significant clustering. Additionally, the S value, which reflects the average contour value of clustering, was determined to be 0.7753. A higher S value closer to 1 indicates greater homogeneity within the clusters.^[25]^ These findings suggest that the clustering results are robust and convincing. The timeline chart of keywords (Fig. 5C) is a view of a change in the clustering of keywords with time, which can reflect the evolution process of hot research topics. On the right is the clustering label, the horizontal axis represents the time. Additionally, the timeline chart of keywords (Fig. 5C) illustrates the evolution of hot research topics over time, with oxidative stress consistently emerging as a prominent research hotspot. Muscle damage garnered significant interest around 2004 but has since declined in popularity, while transcranial magnetic stimulation emerged as an emerging hot spot around 2009 (Fig. 5D). Figure 5E showcases keywords with the strongest citation bursts. Notably, “humans” (2007–2013), “prolonged exercise” (2004–2014), and “physical activity” (2018–2024) exhibit substantial burst intensity values. Recent bursts in “physical activity,” “aerobic exercise,” and “reliability” suggest increasing research interest in understanding the relationship between exercise fatigue, aerobic exercise, and methods for alleviating fatigue. Additionally, “fatigue” emerges as the most central keyword, underscoring its significance within the broader context of fatigue research. Moreover, “electrical stimulation” attracted considerable attention over an 11-year period (2004–2015), indicating its status as a research hotspot during that time. Overall, research primarily focuses on preventing and recovering from sports fatigue, as well as its impact on exercise ability and sports injuries. The relationship between inflammation and exercise has been a hot research topic in recent years in terms of mechanistic studies. Exercise has been increasingly recognized as a beneficial intervention for patients with inflammatory diseases, such as rheumatic diseases, due to its anti-inflammatory and immunomodulatory effects. Regular physical activity has been shown to reduce systemic inflammation, which is a hallmark of rheumatic diseases.^[26]^ Emerging evidence suggests that regular exercise may slow disease progression in some rheumatic conditions by modulating immune responses and reducing chronic inflammation.^[27]^ In conclusion, exercise is a powerful, non-pharmacological intervention for managing inflammatory rheumatic diseases. It not only reduces inflammation and improves physical function but also enhances mental health and overall quality of life. However, individualized approaches are essential to maximize benefits and minimize risks.

### 3.6. Analysis of subject categories and strongest burst keywords

**Figure 6.**
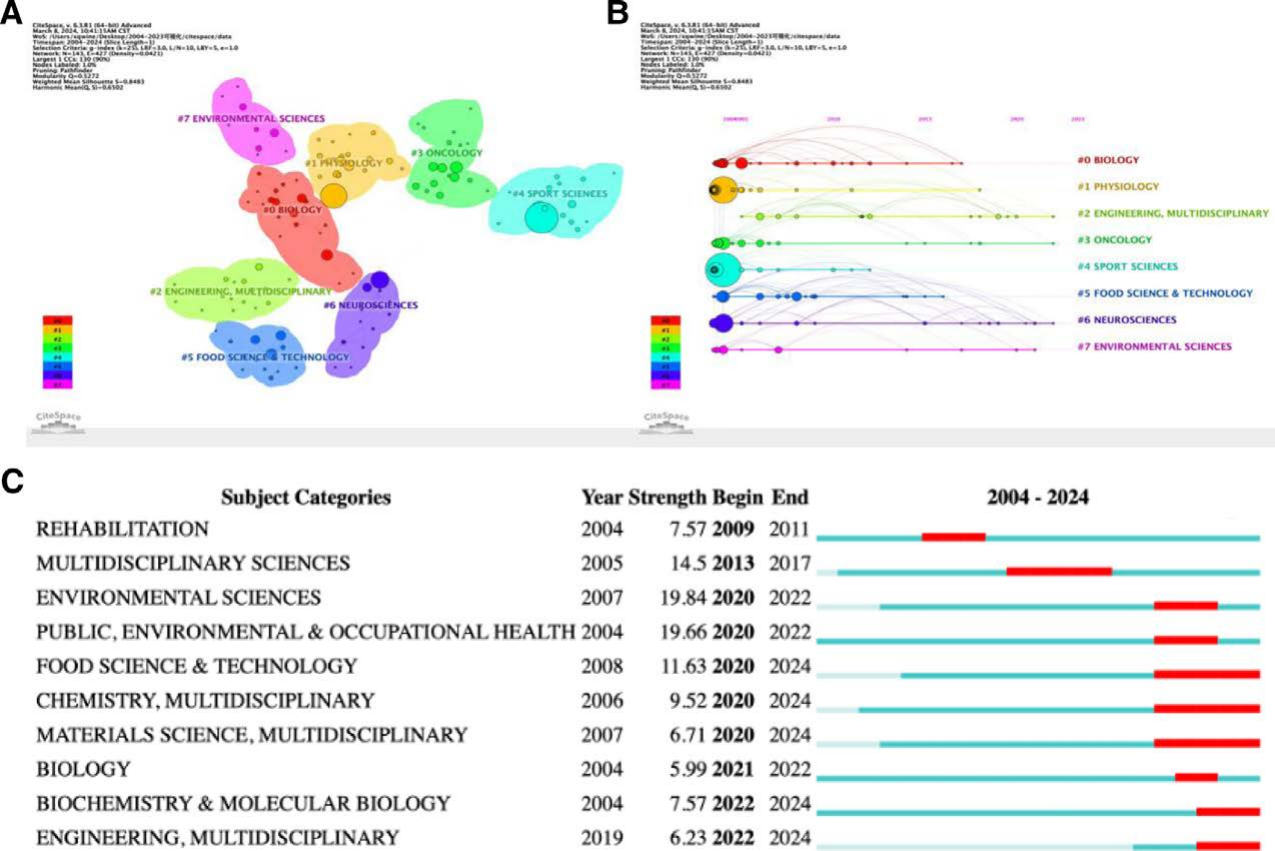
(A) Cluster analysis of subject categories (B) timeline chart of subject categories (C) Top 10 subject categories with the strongest citation bursts.

Since exercise fatigue is widely involved in both kinematics and medicine, visual analysis is carried out for the subject categories of exercise-induced fatigue. A total of 143 different subject categories were identified, with 427 links established for articles on exercise-induced fatigue. Among these, 8 disciplinary categories were selected for analysis: Biology, Physiology, Engineering and Multidisciplinary, Oncology, Sport Sciences, Food Science & Technology, Neuroscience, and Environmental Sciences. From the analysis, it is evident that Biology exhibits the closest relationship with research on sports fatigue, although there has been relatively less research in this area in the past 5 years, possibly due to the existence of a certain research foundation (Fig. 6A). Literature related to Physiology and Sport Sciences predominantly clustered before 2010, suggesting that the combination of exercise fatigue and these disciplines may have been a hot research topic a decade ago (Fig. 6B). There have been notable research results related to the disciplines of Engineering and Multidisciplinary and Oncology, indicating potential current or future research trends. Further in-depth exploration is warranted in these 2 disciplines. Additionally, Food Science & Technology, Chemistry, Materials Science, Biochemistry & Molecular Biology, and Engineering, all under the category of Multidisciplinary, have garnered increased attention in recent years (Fig. 6C). Rehabilitation and Multidisciplinary Sciences were observed to be hot topics in the past but have seen relatively less focus in recent times.

### 3.7. Analysis of journals and strongest burst keywords

**Figure 7.**
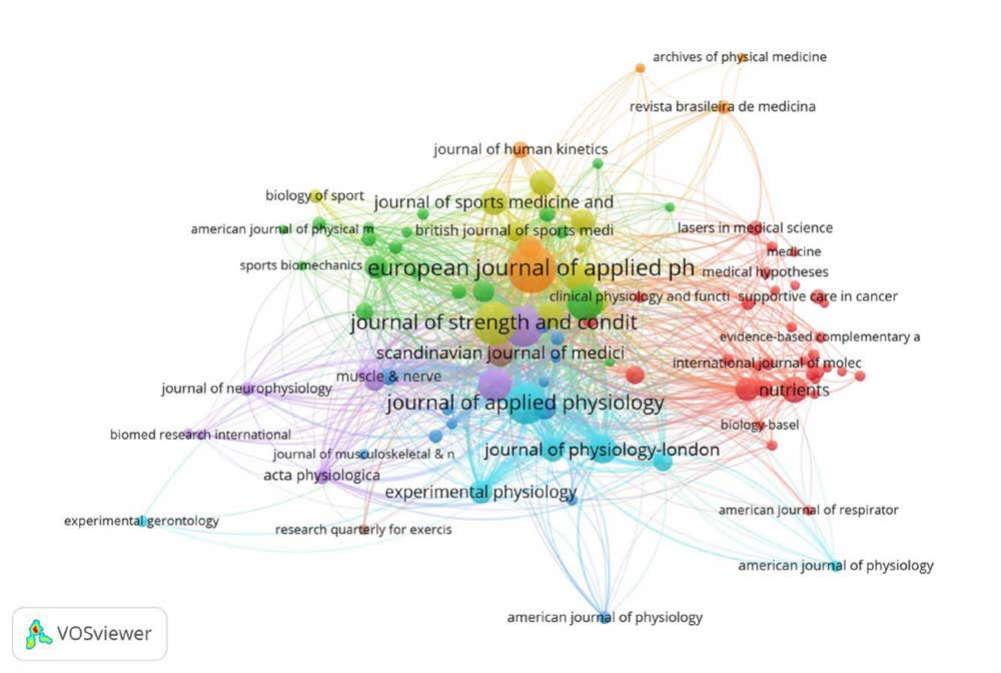
Analysis co-occurrence.

Academic journals serve as a medium for researchers to disseminate their findings and can reflect the quality of research to some extent. In our study, a total of 941 journals were included for analysis. The top 7 cited journals are listed in Table 4, which collectively accounted for 22.93% of the total literature. The journal with the highest total number of citations was Sports Medicine, with 8365 citations and an average citation frequency of 105.89. This was followed closely by the Journal of Applied Physiology (8268 citations) and Sports Medicine and Science (6095 citations). Sports Medicine also boasted the highest impact factor of 7.86, indicating its significant influence in the field. The European Journal of Applied Physiology had the highest number of publications, with 233 articles, comprising 5.1% of the total publications. This suggests that articles in this field are relatively dispersed across various journals. Most journals focusing on exercise-induced fatigue are published in the United States. Figure 7 illustrates a cluster of 8 journals in this field, with different colors representing various disciplines such as medicine, neurology, physiology, kinematics, and human health.

**Table 4:** Top 7 Journals with the highest number of articles on exercise-induced fatigue.

Rank	Journal	Citations	Documents	Impact Factor(2023)	Country	Journal Citation Reports Category
1	European Journal of Applied Physiology	5764	233	2.554	Germany	Q2
2	Journal of Strength and Conditioning Research	4772	176	2.973	United States	Q2
3	Journal of Applied Physiology	8268	164	2.754	United States	Q1
4	Medicine and Science in Sports and Exercise	6095	160	4.478	United States	Q1
5	Frontiers in Physiology	1767	125	4.566	Switzerland	Q1
6	Plos One	2235	102	3.24	United States	Q1
7	Sports Medicine	8365	79	7.86	New Zealand	Q1

### 3.8. Analysis of references

**Figure 8.**
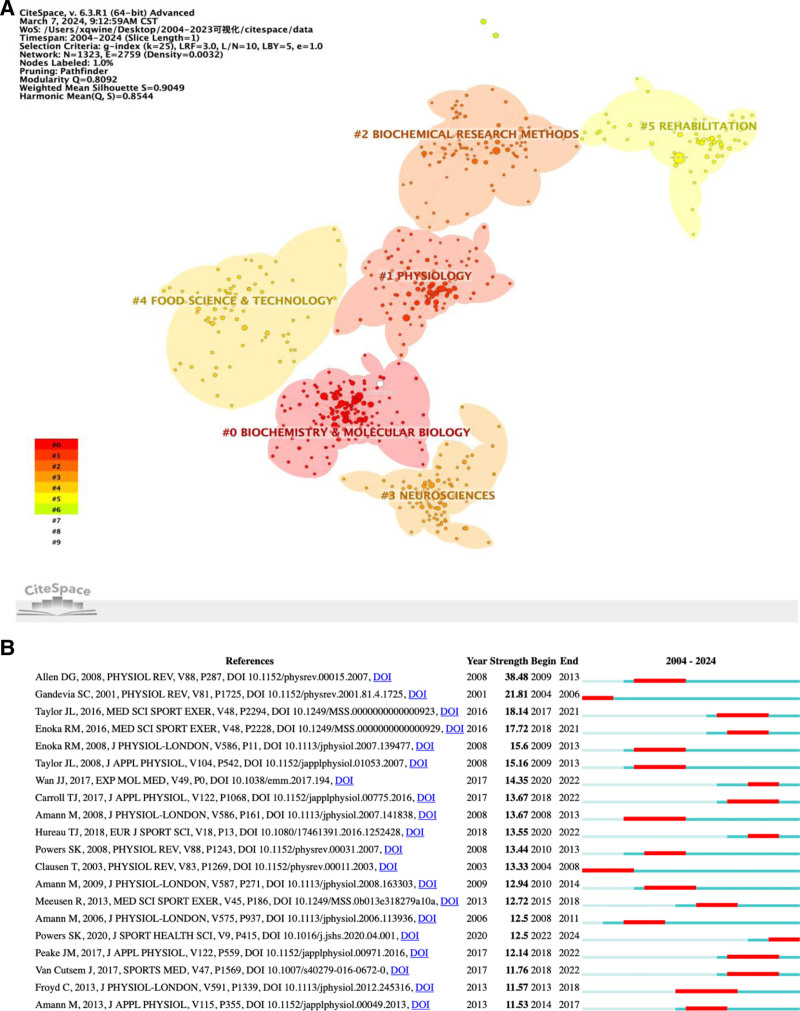
(A) Cluster analysis of references. (B) Top 20 references with the strongest citation bursts.

In bibliometrics, both the number of articles and their citation frequency serve as crucial metrics for assessing influence. Analyzing references helps uncover closely related research topics within the academic field.^[28,29]^ Highly cited papers are indicative of recognized research achievements.^[30]^ Therefore, scrutinizing citation frequency can pinpoint the most influential academic articles, unveiling cutting-edge research hotspots and directions. We included a total of 124,195 cited literature, of which 1323 met the criteria (g-index, LRF, L/N, LBY, e). Figure 8A displays color clusters (numbered 0 to 5) representing Biochemistry & Molecular Biology, Physiology, Biochemical Research Methods, Neurocenters, and Food Science & Technology Rehabilitation, respectively. The citation frequency of an article mirrors its academic stature and impact.^[31]^ Higher citation frequencies denote greater attention received. Table 5 presents the top ten articles based on citation numbers. Notably, the article^[32]^ titled “Exercise-Induced Oxidative Stress: Cellular Mechanisms and Impact on Muscle Force Production” by Scott K Powers and J Jackson garnered the most citations (1570). This article delves into oxidants’ role in sports, addressing the redox modulation of muscle force production/fatigue and redox-sensitive targets within skeletal muscle. Authored by Scott K. Powers and Malcolm J. Jackson from the Department of Applied Physiology and Kinematics, University of Florida, and Division of Metabolic and Cellular Medicine, University of Liverpool, this work significantly contributes to understanding exercise fatigue mechanisms. In Figure 8B, the top 20 references with the strongest citation bursts unveil current research hotspots.^[33]^ Analyzing these references aids in predicting future research trends. Notably, an article published in the Journal of Sport and Health Science titled “Exercise-induced oxidative stress: Friend or foe?”^[34]^ has garnered continuous attention since its 2020 publication. The highest Strength Index was observed in a 2008 article titled “Skeletal Muscle Fatigue: Cellular Mechanisms,”^[1]^ which had a substantial impact from 2009 to 2014. These articles with robust citation bursts epitomize research hotspots of their time, exploring exercise-induced fatigue mechanisms from various angles.

**Table 5:** Top 10 articles with the highest number of citations on exercise-induced fatigue.

Rank	Article	Journal	Citations	IF (2023)	Publication year
1	Exercise-Induced Oxidative Stress: Cellular Mechanisms and Impact on Muscle Force Production	Physiological Reviews	1570	28.312	2008
2	Lactate metabolism: a new paradigm for the third millennium	The Journal of Physiology	818	5.5	2004
3	Biochemistry of exercise-induced metabolic acidosis	Physiology-regulatory Integrative and Comparative Physiology	788	1.867	2004
4	The effect of exercise-induced arousal on cognitive task performance: A meta-regression analysis	Brain Research	700	2.307	2010
5	Oxidative Stress	Sports Medicine	563	7.86	2012
6	Repeated-Sprint Ability – Part I	Sports Medicine	550	7.86	2012
7	Six sessions of sprint interval training increases muscle oxidative potential and cycle endurance capacity in humans	Journal of Applied Physiology	540	2.754	2008
8	Recruitment Patterns in Human Skeletal Muscle During Electrical Stimulation	Physical Therapy	400	3.87	2005
9	Potential health-promoting effects of astaxanthin: A high-value carotenoid mostly from microalgae	Molecular Nutrition & Food Research	393	3.938	2011
10	A comparison of central aspects of fatigue in submaximal and maximal voluntary contractions	Journal of Applied Physiology	382	2.754	2008

## 4. Future directions

After visual analysis of the research from 2004 to 2023, it is evident that the number of papers on exercise-induced fatigue has experienced a sudden increase and is now in a state of reduction. Scholars in this field should persist in seeking breakthroughs to address prevailing challenges. The research on exercise-induced fatigue is dominated by the United States, with many other countries collaborating, showcasing a positive trend. However, there’s a need to bolster inter-country cooperation to yield more cutting-edge results. Strengthening collaboration between institutions and authors is also imperative to enhance research quality. From the keywords of research hotspots, we can see that the current research focuses on the prevention and recovery of exercise-induced fatigue, as well as sports ability and sports injury. As for the physiological mechanism of exercise-induced fatigue, although some studies have linked exercise-induced fatigue to stress response,^[35,36]^ metabolic regulation,^[37,38]^ neurotransmitter changes, energy metabolism imbalance, internal environment disturbances, neural regulation,^[39–41]^ oxidative stress,^[42,43]^ and activity-related transcription factors,^[44,45]^ the exact molecular mechanisms remain incompletely understood. Future research should continue to focus on determining the specific mechanisms of exercise-induced fatigue, thus providing an objective basis for establishing pathological diagnostic criteria, although this has so far been a difficult problem. In addition, skeletal muscle, as a direct participant in exercise, has been closely linked to the study of exercise-induced fatigue. Multi-omics techniques enable accurate screening analysis of exercise-related specific biomarkers,^[46]^ revealing biomarkers of peripheral muscle fatigue that are important for monitoring and in-depth analysis of the mechanisms underlying the occurrence and development of exercise-induced fatigue.^[47]^ Additionally, oxidative stress emerges as a significant factor in the study of exercise-induced fatigue. Oxidative stress was defined as antioxidants and oxidants imbalance in favor of the oxidants, which potentially leads to deterioration.^[48,49]^ Studies have shown that prolonged, high-intensity exercise can lead to excessive lactic acid supply to glial cells, resulting in oxidative stress in peripheral axonal neurons,^[50]^ ultimately leading to axonal degeneration and disruption of peripheral axon integrity.^[51]^ This novel finding may offer new insights into elucidating the mechanism of exercise-induced fatigue. Based on the elucidation of the mechanism of exercise-induced fatigue, it is necessary to further screen specific activators of natural activity against exercise-induced fatigue, so as to provide potential safe and effective methods and approaches for intervention.^[52,53]^ At the same time, the impact of exercise-induced fatigue on patients ‘psychological and emotional health deserves attention and is also a key area that needs further exploration. Recognizing the intrinsic connection between physical health and mental equilibrium, integrating psychological aspects into treatment regimens holds the potential for the incidence of exercise-induced fatigue, and related psychological interventions need to be explored.^[54]^ Concurrently, the shortage of relevant clinical trial studies underscores the necessity for continued high-quality clinical trials to provide a solid foundation for clinical application. With the continuous progress of medical science and technology, the research on exercise-induced fatigue is not only limited to sports medicine, but also intersects with biology, physiology, neurology and other disciplines. This cooperation is expected to unlock the complexity of exercise-induced fatigue and provide accurate diagnosis and effective treatment options for patients. It is imperative to strengthen multidisciplinary intersections in the future to facilitate further advancements in this field. Finally, exploring other analytical tools such as machine learning techniques in bibliometric analysis could improve the efficiency and accuracy of future reviews.

## 5. Strength and limitations

This article represents the inaugural endeavor to conduct bibliometric and visual analysis on sports fatigue. This innovative analytical approach facilitates a comprehensive understanding of the research focus and hotspots in this domain over the past 2 decades. For clinicians, this review provides a comprehensive overview of the current research landscape on exercise-induced fatigue, informing clinical decision-making and treatment planning. For researchers, it identifies gaps in the literature and potential areas for future investigation. For patients, it raises awareness about the multifactorial nature of fatigue and promotes personalized management strategies. Nevertheless, our analysis is subject to certain limitations. Firstly, our study exclusively relied on the Web of Science Core Collection as the literature database, thereby omitting potential insights from other databases such as Scopus. This limitation might have restricted the comprehensiveness of our analysis. As a result, some of the most recent research on exercise-induced fatigue may not have been included in our analysis. We acknowledge this limitation and recommend that future updates to this review incorporate data from the latest publications. Additionally, our analysis was confined to English-language research indexed in Web of Science Core Collection, potentially overlooking valuable contributions from non-English sources. Moreover, the fixed retrieval time poses a challenge, as citation frequencies post-retrieval cannot be updated, potentially introducing bias into our findings. To maintain focus and consistency, our review was limited to studies published within a specific time range. While this allowed us to analyze trends within a defined period, it may have excluded earlier foundational studies or very recent breakthroughs. Future studies could expand the time frame to capture a more comprehensive picture of the field. Future studies could explore longer time frames or specific sub-periods to further validate and extend our findings. Software limitations also constrained our analysis, preventing the modification of capitalization and abbreviations. Additionally, setting thresholds and cropping methods may have inadvertently led to data truncation, potentially affecting the accuracy of our results. Addressing these limitations in future studies will be crucial for enhancing the comprehensiveness and accuracy of bibliometric analyses in the field of sports fatigue.

## 6. Conclusion

Exercise-induced fatigue is attracting more and more attention from scholars. This study analyzed the research on exercise-induced fatigue during the 20 years from January 2004 to December 2019 by bibliometrics tools, and reviewed the history of this field from many aspects including research country, institution, author, discipline, journal, cited and key words. At present, a lot of research focuses on the mechanism of exercise-induced fatigue. Although there is a certain research basis, there is still a lack of high-quality evidence-based medical evidence. In the future, efforts should be dedicated to improve the quality of research in the field of exercise-induced fatigue to clarify the mechanism of exercise-induced fatigue, find objective criteria for measuring the incidence of exercise-induced fatigue and take measures to alleviate it. This will involve conducting rigorous and methodologically sound studies, including randomized controlled trials, matched sample cross-sectional studies, and meta-analyses. Exercise-induced fatigue is a multifaceted phenomenon that intersects with physiology, psychology, neuroscience, and sports science. Future research could benefit from more interdisciplinary collaborations to address complex questions and develop holistic interventions. With the rapid development of technologies such as wearable devices, artificial intelligence, and advanced imaging techniques, future reviews could explore how these tools are being applied to study exercise-induced fatigue. Future research should prioritize translating findings into practical applications, such as developing evidence-based guidelines for fatigue management in athletes, patients, and the general population. This could include personalized training programs, nutritional interventions, and recovery strategies. This could shed light on novel methodologies and their impact on the field. By understanding the current status and future trajectory of exercise-induced fatigue research, we hope to provide some insights and ideas for this research field, thus guiding research direction and clinical application.

## Acknowledgments

These authors would like to thank the software developers of VOSviewer and CiteSpace. Thank you to the database for providing data information.

## Author contributions

**Conceptualization:** Qiwen Xuan.

**Project administration:** Wei Gu.

**Visualization:** Qiwen Xuan, Lele Huang.

**Writing – original draft:** Qiwen Xuan, Lele Huang.

**Writing – review & editing:** Wei Gu, Changquan Ling.
